# Local knowledge and exploitation of the avian fauna by a rural community in the semi-arid zone of northeastern Brazil

**DOI:** 10.1186/1746-4269-10-81

**Published:** 2014-12-24

**Authors:** Pedro Hudson Rodrigues Teixeira, Thiago do Nascimento Thel, Jullio Marques Rocha Ferreira, Severino Mendes de Azevedo, Wallace Rodrigues Telino Junior, Rachel Maria Lyra-Neves

**Affiliations:** Departamento de Biologia, Programa de Pós-graduação em Ecologia, Universidade Federal Rural de Pernambuco – UFRPE, Rua Dom Manoel de Medeiros, s/n, Dois Irmãos, Recife/PE, CEP: 52171-900 Brasil

**Keywords:** Ethnozoology, Ethno-ornithology, Hunting, Zootherapy

## Abstract

**Background:**

The present study examined the exploitation of bird species by the residents of a rural community in the Brazilian semi-arid zone, and their preferences for species with different characteristics.

**Methods:**

The 24 informants were identified using the “snowball” approach, and were interviewed using semi-structured questionnaires and check-sheets for the collection of data on their relationship with the bird species that occur in the region. The characteristics that most attract the attention of the interviewees were the song and the coloration of the plumage of a bird, as well as its body size, which determines its potential as a game species, given that hunting is an important activity in the region.

**Results:**

A total of 98 species representing 32 families (50.7% of the species known to occur in the region) were reported during interviews, being used for meat, pets, and medicinal purposes. Three species were used as zootherapeutics – White-naped Jay was eaten whole as a cure for speech problems, the feathers of Yellow-legged Tinamou were used for snakebite, Smooth-billed Ani was eaten for “chronic cough” and Small-billed Tinamou and Tataupa Tinamou used for locomotion problems. The preference of the informants for characteristics such as birdsong and colorful plumage was a significant determinant of their preference for the species exploited. Birds with cynegetic potential and high use values were also among the most preferred species. Despite the highly significant preferences for certain species, some birds, such as those of the families Trochilidae, Thamnophilidae, and Tyrannidae are hunted randomly, independently of their attributes.

**Conclusion:**

The evidence collected on the criteria applied by local specialists for the exploitation of the bird fauna permitted the identification of the species that suffer hunting pressure, providing guidelines for the development of conservation and management strategies that will guarantee the long-term survival of the populations of these bird species in the region.

## Background

Human beings have always exploited wild animals for a variety of resources, and their relationship with these animals can be observed in the hunting scenes found in ancient rock paintings in South America [1, 2]. In recent years, a scientific discipline, known as Ethnozoology, has been developed to provide a systematic understanding of the different types of interaction between humans and animals [3].

A number of different subsistence traditions can be found in the human populations of the Brazilian semi-arid zone, including hunting-and-gathering strategies. These strategies are supported by empirical knowledge on the most efficient practices for the acquisition and use of natural resources accumulated over many generations [4].

Worldwide, birds represent one of the most important groups of vertebrates that are hunted for food, and have attracted the attention of humans in a number of different ways over recorded history [2]. Birds present a number of characteristics, such as complex vocalizations and vividly-colored plumage, which not only makes them attractive to humans, but also permits the reliable identification of species in the wild [5, 6]. The various interactions between humans and birds, and local knowledge of this fauna are of considerable relevance for the conservation of the avian fauna of a given area, and ethno-ornithology is a fundamentally important tool for the gathering of such information [4, 7, 8].

Studies of the exploitation of wild animals – including birds – by human populations have been conducted in many regions of the world [3, 4, 9–12], providing information on the diversity of the fauna used and the patterns of exploitation of these animals by traditional rural communities. In addition to subsistence hunting [13, 14] and the raised in captivity of birds for pets and illegal trade, a number of studies have reported the medicinal use of some species [9–12].

In Brazil, the exploitation of the native fauna is intensified in areas such as the semi-arid Northeast, which has a population of some 28 million inhabitants, many of whom depend on locally-available natural resources, given the adverse environmental conditions found throughout most of the region [4]. In the specific case of the birds, interactions with human populations include subsistence hunting, pets, and illegal trade [8, 15–17], in addition to the occasional use of some species for medicinal purposes [4, 18–25].

In the Brazilian Northeast, 108,041 wild birds were confiscated from illegal traders by government agencies between 1992 and 2000 [26], far more than in any other part of the country. Birds represent the primary focus of the illegal trade in wild animals in Brazil, representing 82% of the 36,370 animals confiscated in the country in 1999 and 2000. These values reflect the clear impacts on the country’s natural ecosystems. Most of the species captured for the illegal trade are songbirds, in particular emberezids, or have exuberant plumage, such as psittacids, thraupids, and cardinalids [8, 16].

The Araripe National Forest has a diverse avian fauna. Nascimento et al. [27] produced an updated inventory of 193 bird species for the area. Many of the species known to occur in the area present potentially attractive characteristics, such as the seedeater birds (*Sporophila* spp., *Sicalis* spp. and *Cyanoloxia brissonii*), which are songbirds, and in the latter two cases, have exuberant plumage, which is also a characteristic of species such as the tanagers, parakeets and parrots. Some of the region’s bird species are endemic and/or endangered with extinction [28], including those targeted by subsistence hunters, such as the guans and tinamous (*Penelope* spp. Tinamidae), and illegal traders, in particular the Yellow-faced Siskin (*Sporonga yarrellii*), but above all, the Araripe Manakin (*Antilophia bokermanni*), a species that is endemic to the Araripe NF, and is classified as critically endangered [29] due primarily to the loss of habitats.

Given these considerations, the present study focused on the bird fauna of a region in the Brazilian semi-arid zone, with the primary aim of identifying the principal factors that determine the selection of the species exploited by the local residents. The study tests the hypothesis that colorful plumage and attractive birdsong are the principal attributes appreciated by the local informants, and that the species with these attributes and a good hunting potential are the most important to the local community. The study focused on the avian fauna of the Chapada do Araripe, and the principal characteristics used by local hunters as criteria for the selection of birds hunted for game or captured for other uses.

## Material and methods

### Study area

The Araripe National Forest is a sustainable use conservation unit located within the Araripe Environment Protection Area (APA Araripe) which was created by Brazilian federal decree number 9226/46. The APA Araripe, which is also a sustainable use protected area in the Brazilian semi-arid zone, covers approximately 1,063,000 hectares and was created on August 4th, 1997 [30]. This area includes parts of the Brazilian states of Ceará, Pernambuco, and Piauí.

**Figure 1:**
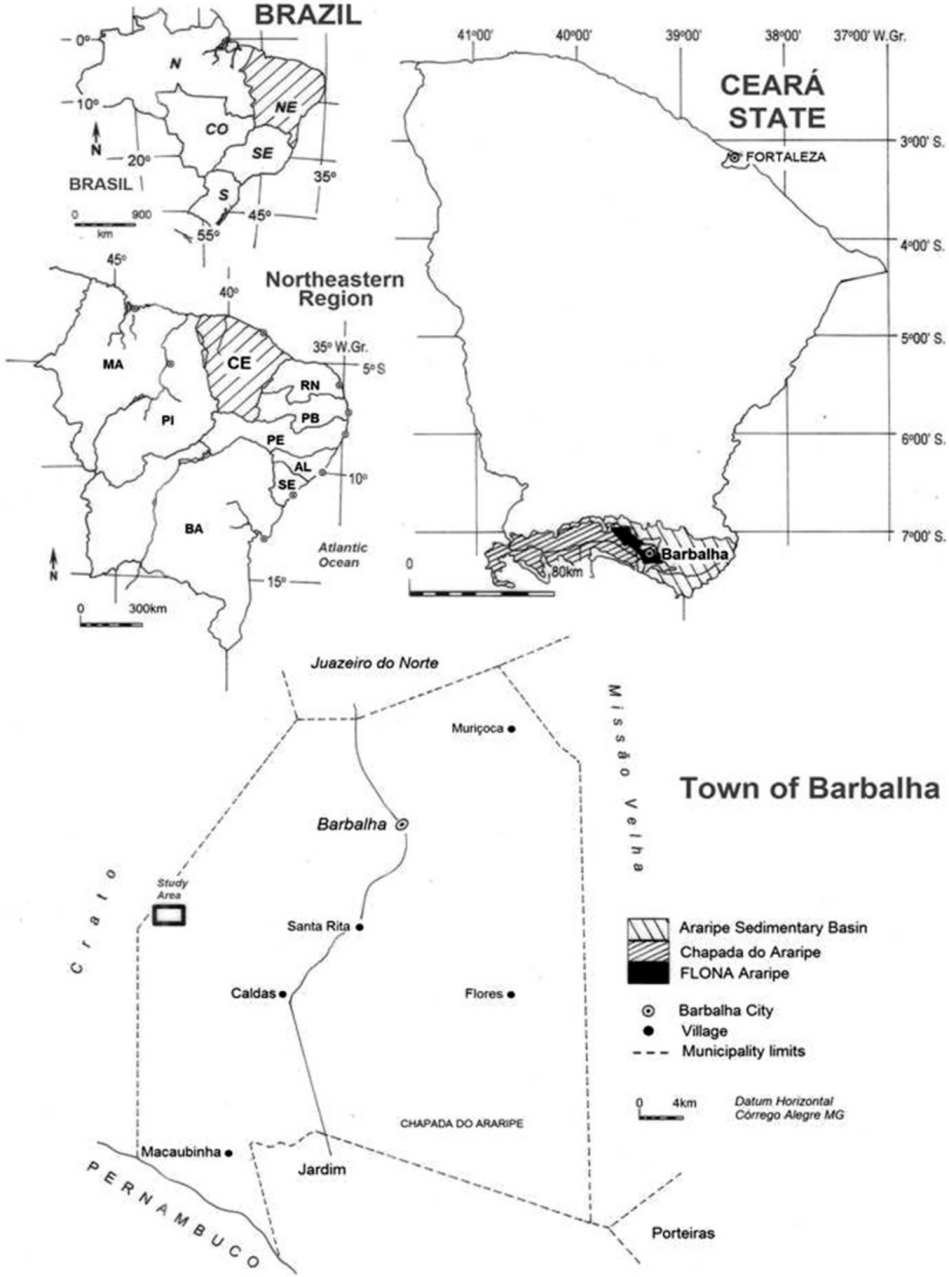
**Location of the study area, Macaúba, in the municipality of Barbalha, in the northeastern Brazilian state of Ceará.**

The Araripe NF is located in the eastern extreme of the Araripe Plateau, including areas of the municipalities of Crato, Barbalha, and Jardim, and is considered to be the first Brazilian national forest [29]. The relief is flat, with a mean altitude of 800 m a.s.l. Mean annual precipitation is 1100 mm, with mean temperatures of between 15°C and 25°C. Vegetation types include seasonal semi-deciduous forest, cerrado savanna, savanna woodland, and scrub [31].The rural community of Macaúba is located within the APA Araripe (Figure 1), in the municipality of Barbalha (Brazilian state of Ceará), some 610 km from the state capital, Fortaleza. The community contains approximately 250 families, and most of the residents are farmers who also harvest plant species occurring naturally within the area of the Araripe NF.

Macaúba was selected for the present study based on information provided by local technicians of the federal environment protection agencies (IBAMA and ICMBio) who reported high rates of environmental crime in the region, in particular the hunting and capture of wild animals. In addition, the management plan for the conservation unit refers specifically to the traditional hunting practices of this community [29].

### Data collection and analysis

Field trips to Macaúba began with conversations with local residents who could indicate possible specialist informants within the community who were known to exploit wild birds in some way. These initial interviewees included the local public health agents and the president of the Macaúba residents association, individuals who had contact with all the local families. Specialists were the residents known to have the greatest knowledge on the local avian fauna and capture techniques, as well as being proficient hunters of birds. A total of 24 interviews were conducted between September, 2012, May, 2013 and November, 2014.

Following this initial contact with local residents, specialists were identified using the “snowball” technique [32], in which informants are selected through the identification and interviewing of specialists, who are asked to indicate other specialists, who are also interviewed, and so on successively until the names cited begin to be repeated, indicating that further interviews are unnecessary. Preliminary contacts with residents from the Macaúba community indicated the first interviewees, who in turn indicated all other informants. A number of residents hunt and capture animals, although the specific objective of the study was to obtain information on the exploitation of bird species, which led to the discarding of many interviews, given that most informants only referred to the use of mammals, such as deer, agoutis, cats, and monkeys, or no longer practiced hunting. This led to the selection of 24 residents, who constituted the complete sample universe, and were considered to be specialists on the region’s birds and the strategies used to hunt and capture the different species.

The local knowledge of all the informants was also investigated using semi-structured interviews [33] in order to understand the characteristics of the bird species – such as vocalizations or plumage – that determine the specialists’ preferences or choices, based on their knowledge and experience of the region’s avian fauna. All the interviews were recorded with the consent of the informants.

Each interviewee was informed *a priori* of the objectives of the research and asked to sign a standard informed consent form. The present study was approved by the Brazilian System for the Inventory and Authorization of National Biodiversity (SISBIO) under license number 30533–2, as well as the Ethics in Research Committee of the Federal Rural University of Pernambuco (Protocol CAAE 08413112.5.0000.5207).

At the end of each interview, the informant was invited to review a check-sheet of photographs showing bird species known to occur in the region [27], as well as seven species that are not found in the study area, which were used as a control for the evaluation of the reliability of the information provided in the interviews. The informants were asked to point to the species they hunted for meat, raised for captivity or used for medicinal purposes.

A use-value or UV [34] was calculated for each bird species, providing an index of relative importance of the species to the local human population. The UV score is calculated for a species is given by UV = ΣU/n, where ΣU = the species was cited by an informant (for one or both uses) and n = the total number of informants.

## Results

Two categories of exploitation were identified. One was hunting for game meat or medicinal purposes and the other, capture for pets. Fifteen of the informants reported exploiting birds for both game and pets, seven reported hunting for game, six for medicinal purposes, and three for pets. Based on the check-sheets, the informants confirmed using 97 species belonging to 30 families, which represent 33.5% of the bird species known to occur in the Araripe NF (Table 1).

**Table 1:** **Bird species cited by the informants interviewed at Macaúba, Barbalha, Ceará, northeastern Brazil**

Family	Species	Brazilian common name	Local common name	Use		Total	UV
				Game	Pets		
Tinamidae	*Crypturellus noctivagus*	Yellow-legged Tinamou	Zabelê	8		8	0.33
	*Crypturellus parvirostris*	Small-billed Tinamou	Nambu	15		15	**0.63**
	*Crypturellus tataupa*	Tataupa Tinamou	Nambu	16		16	**0.70**
	*Nothura maculosa*	Spotted Nothura	Corduniz	12		12	**0.50**
Cracidae	*Penelope superciliaris*	Rusty-margined Guan	Jacu	18	3	21	**0.90**
Accipitridae	*Rupornis magnirostris*	Roadside Hawk	Gavião	3		3	0.12
Columbidae	*Columbina minuta*	Plain-breasted Ground-Dove	Rolinha	5	1	6	0.25
	*Columbina talpacoti*	Ruddy Ground-Dove	Rolinha- caldo-de-feijão	7	1	8	0.33
	*Columbina squammata*	Scaled Dove	Fogo-apagou	4	5	9	0.37
	*Columbina picui*	Picui Ground-Dove	Rolinha	8		8	0.33
	*Claravis pretiosa*	Blue Ground-Dove	Rola-azul	3		3	0.12
	*Leptotila verreauxi*	White-tipped Dove	Juriti	10	1	11	0.45
Cuculidae	*Piaya cayana*	Squirrel Cuckoo	Alma-de-gato	2		2	0.08
	*Crotophaga ani*	Smooth-billed Ani	Anu-preto	3		3	0.12
	*Guira guira*	Guira Cuckoo	Anu-branco	1		1	0.04
	*Tapera naevia*	Striped Cuckoo	Saci	1		1	0.04
Strigidae	*Glaucidium brasilianum*	Ferruginous Pygmy-Owl	Caboré	1		1	0.04
Nyctibiidae	*Nyctibius griseus*	Common Potoo	Mãe-da-lua	2		2	0.08
Caprimulgidae	*Hydropsalis albicollis*	Pauraque	Corujinha	3		3	0.12
Trochilidae	*Anopetia gounellei*	Broad-tipped Hermit		1		1	0.04
	*Phaethornis pretrei*	Planalto Hermit		2		2	0.08
	*Eupetomena macroura*	Swallow-tailed Hummingbird	Tesorão	4		4	0.16
	*Thalurania watertonii*	Long-tailed Woodnymph	Bizunguinha	1		1	0.04
	*Amazilia fimbriata*	Glittering-throated Emerald	Bizunga	4		4	0.16
Trogonidae	*Trogon curucui*	Blue-crowned Trogon	Cizuda	2		2	0.08
Galbulidae	*Galbula ruficauda*	Rufous-tailed Jacamar	Fura-barreira	2		2	0.08
Picidae	*Veniliornis passerines*	Little Woodpecker	Picapauzinho	1		1	0.04
	*Piculus chrysochloros*	Golden-green Woodpecker	Pica-pau	2		2	0.08
	*Celeus flavescens*	Blond-crested Woodpecker	Pica-pau-amarelo	2		2	0.08
Cariamidae	*Cariama cristata*	Red-legged Seriema	Sariema	5	1	6	0.25
Falconidae	*Falco femoralis*	Aplomado Falcon	Gavião		1	1	0.04
Psittacidae	*Eupsittula cactorum*	Cactus Parakeet	Ganguirro		11	11	**0.45**
	*Forpus xanthopterygius*	Blue-winged Parrotlet	Pancu	1	2	3	0.12
	*Amazona aestiva*	Blue-fronted Parrot	Papagaio	3	1	4	0.16
Thamnophilidae	*Myrmorchilus strigilatus*	Stripe-backed Antbird	Piu-piu	5		5	0.20
	*Formicivora grisea*	White-fringed Antwren	Papa-formiga	5		5	0.20
	*Formicivora melanogaster*	Black-bellied Antwren	Papa-formiga	4		4	0.16
	*Dysithamnus mentalis*	Plain Antvireo		1		1	0.04
	*Herpsilochmus atricapillus*	Black-capped Antwren		2		2	0.08
	*Herpsilochmus longirostris*	Large-billed Antwren	Chapeuzinho	3		3	0.12
	*Sakesphorus cristatus*	Silvery-cheeked Antshrike	Farinheiro	7		7	0.29
	*Thamnophilus capistratus*	Caatinga Antshrike	Rajado	10		10	**0.41**
	*Thamnophilus torquatus*	Rufous-winged Antshrike		4		4	0.16
	*Thamnophilus punctatus*	Northern Slaty-Antshrike		4		4	0.16
	*Taraba major*	Great Antshrike	Chorró-olho-de-fogo	5		5	0.20
Scleruridae	*Sclerurus scansor*	Rufous-breasted Leaftosser	Vira-folha	1			0.04
Dendrocolaptidae	*Lepidocolaptes angustirostris*	Narrow-billed Woodcreeper	Arapaçu	1		1	0.04
Furnariidae	*Phacellodomus rufifrons*	Rufous-fronted Thornbird	João-graveteiro	1		1	0.04
Tityridae	*Myiobius atricaudus*	Black-tailed Flycatcher		2		2	0.08
Rhynchocyclidae	*Leptopogon amaurocephalus*	Sepia-capped Flycatcher		1		1	0.04
	*Hemitriccus margaritaceiventer*	Pearly-vented Tody-tyrant	Relojinho	1		1	0.04
Tyrannidae	*Euscarthmus meloryphus*	Tawny-crowned Pygmy-Tyrant	Doidinha	2		2	0.08
	*Elaenia flavogaster*	Yellow-bellied Elaenia	Doidinha	1		1	0.04
	*Elaenia parvirostris*	Small-billed Elaenia	Doidinha	1		1	0.04
	*Elaenia mesoleuca*	Olivaceous Elaenia	Doidinha	3		3	0.12
	*Elaenia cristata*	Plain-crested Elaenia	Doidinha	3		3	0.12
	*Elaenia chiriquensis*	Lesser Elaenia	Doidinha	4		4	0.16
	*Myiopagis caniceps*	Gray Elaenia		1		1	0.04
	*Myiopagis viridicata*	Greenish Elaenia		1		1	0.04
	*Legatus leucophaius*	Piratic Flycatcher		3		3	0.12
	*Myiarchus swainsoni*	Swainson’s Flycatcher	Cacuruta	3		3	0.12
	*Myiarchus ferox*	Short-crested Flycatcher		5		5	0.20
	*Myiarchus tyrannulus*	Brown-crested Flycatcher		5		5	0.20
	*Pitangus sulphuratus*	Great Kiskadee	Bem-ti-vi	1		1	0.04
	*Myiodynastes maculates*	Streaked Flycatcher	Rajado	12		12	**0.50**
	*Megarynchus pitangua*	Boat-billed Flycatcher	Neinei	2		2	0.08
	*Tyrannus melancholicus*	Tropical Kingbird	Burraiera	3		3	0.12
	*Empidonomus varius*	Variegated Flycatcher	Sujinha	9		9	**0.37**
	*Myiophobus fasciatus*	Bran-colored Flycatcher		2		2	0.08
	*Cnemotriccus fuscatus*	Fuscous Flycatcher		1		1	0.04
	*Lathrotriccus euleri*	Euler’s Flycatcher		2		2	0.08
Corvidae	*Cyanocorax cyanopogon*	White-naped Jay	Cancão	6	7	13	**0.54**
Polioptilidae	*Polioptila plumbea*	Tropical Gnatcatcher		1		1	0.04
Turdidae	*Turdus rufiventris*	Rufous-bellied Thrush	Sabiá-laranjeira		4	4	0.16
	*Turdus leucomelas*	Pale-breasted Thrush	Sabiá-branca	1	6	7	0.29
	*Turdus amaurochalinus*	Creamy-bellied Thrush	Sabiá-bico-de-osso	3	5	8	0.33
	*Turdus albicollis*	White-necked Thrush	Sabiá-coleira		1	1	0.04
Mimidae	*Mimus saturninus*	Chalk-browed Mockingbird	Sabiá-conga	1		1	0.04
Thraupidae	*Coereba flaveola*	Bananaquit	Cambacica	1		1	0.04
	*Lanio pileatus*	Pileated Finch	Abre-e-fecha		3	3	0.12
	*Lanio cucullatus*	Red-crested Finch	Abre-e-fecha		1	1	0.04
	*Tangara sayaca*	Sayaca Tanager	Sanhaçu	3	2	2	0.08
	*Tangara palmarum*	Palm Tanager	Sanhaçu-coqueiro	1	1	2	0.08
	*Tangara cayana*	Burnished-buff Tanager	Saíra-amarela	2	2	4	0.16
	*Paroaria dominicana*	Red-cowled Cardinal	Cabeça-vermelha		8	8	0.33
	*Dacnis cayana*	Blue Dacnis	Sanhaçu-azul		1	1	0.04
	*Schistochlamys ruficapillus*	Cinnamon Tanager			1	1	0.04
	*Zonotrichia capensis*	Rufous-collared Sparrow	Tico-tico	1	3	4	0.16
	*Sicalis flaveola*	Saffron Finch	Canário-verdadeiro		12	12	**0.50**
	*Sporophila lineola*	Lined Seedeater	Bigodeiro		4	4	0.16
	*Sporophila albogularis*	White-throated Seedeater	Golinha		5	5	0.20
	*Sporophila nigricollis*	Yellow-bellied Seedeater	Bico-de-prata		5	5	0.20
Cardinalidae	*Cyanoloxia brissonii*	Ultramarine Grosbeak	Azulão		4	4	0.16
Icteridae	*Icterus pyrrhopterus*	Variable Oriole	Viana		3	3	0.12
	*Icterus jamacaii*	Campo Troupial	Sofreu ou Sofrê		4	4	0.16
Fringillidae	*Sporagra yarrellii*	Yellow-faced Siskin	Pintasilva		1	1	0.04
	*Euphonia chlorotica*	Purple-throated Euphonia	Vim-vim		1	1	0.04

UV = Use Value. Nomenclature based on CBRO. In bold type: species characterized by their high use value.

The song was the characteristic most mentioned by the interviewees, being cited by all the informants, followed by colorful plumage, mentioned by 14 informants. Almost all the informants referred to the potential of the birds as game. It was possible to confirm that the interviewees generally based their selection of bird species on characteristics judged to be attractive. Regarding the use of meat for consumption, the species that had the VU > 0.30, 16 (62,5%) have hunting potential. However, 64 (95,3%) species with hunting potential had the VU <0.30.

**Figure 2:**
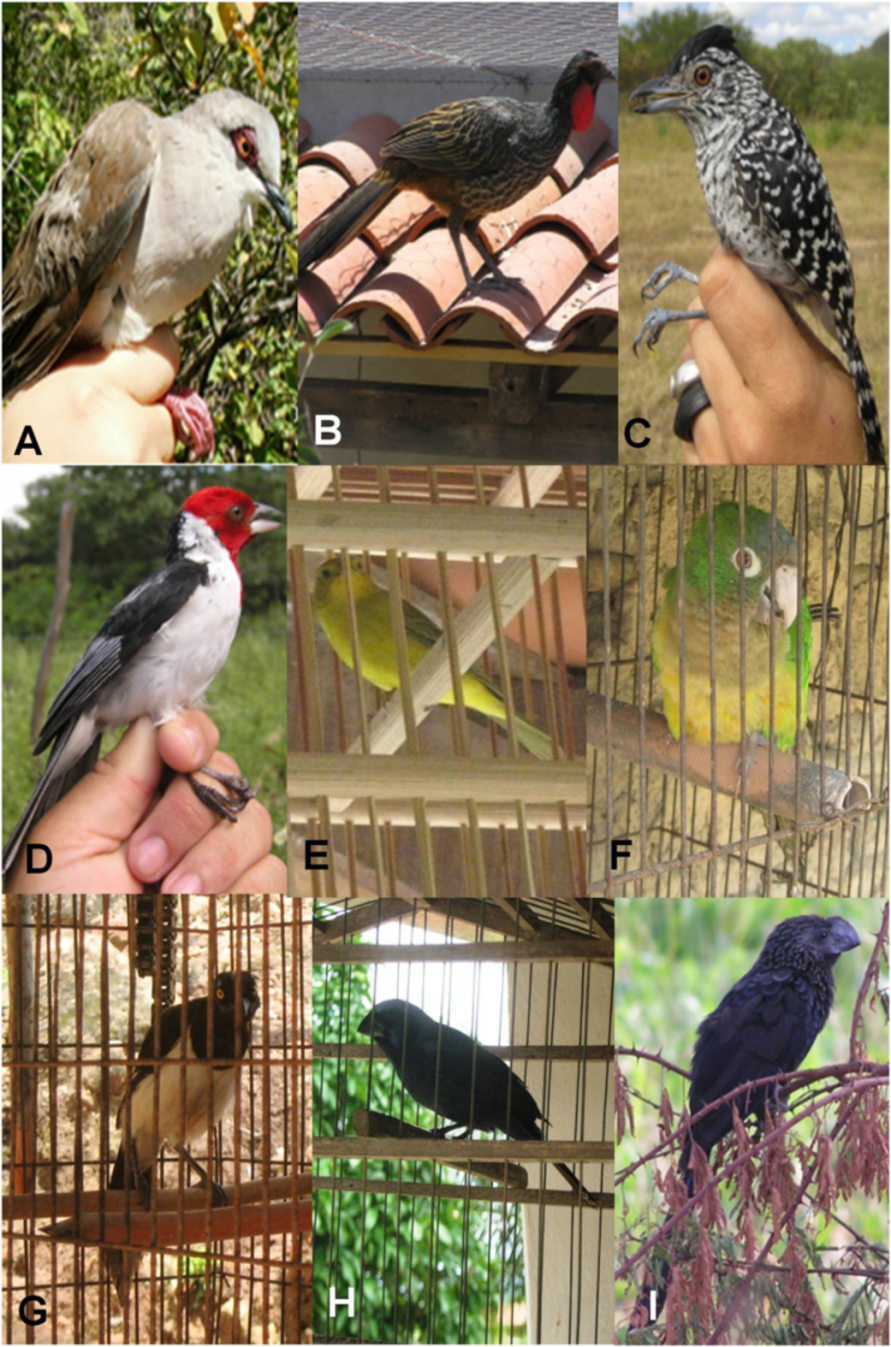
**Some of the bird species captured by residents of Macaúba, Barbalha, Ceará for their meat: A –** ***Leptotila verreauxi*** **; B -** ***Penelope superciliaris*** **; C -** ***Thamnophilus capistratus*** **; D -** ***Paroaria dominicana*** **; E -** ***Sicalis flaveola*** **; F –** ***Eupsittula cactorum*** **; G –** ***Cyanocorax cyanopogon*** **; H –** ***Cyanoloxia brissonii*** **; I –** ***Crotophaga ani*** **.**

The most important game species belong to five families – the Tinamidae (*Crypturellus parvirostris* with 15 reports and *C. tataupa* with 16), Cracidae (*Penelope superciliaris*, n = 21 reports), Columbidae (*Columbina picui*, n = 8; *Leptotila verreauxi*, n = 11), Thamnophilidae (*Sakesphorus cristatus*, n = 7; *Thamnophilus capistratus*, n = 10), and the Tyrannidae (*Myiodinastes maculatus*, n = 12; *Empidonomus varius*, n = 9). Species of five families were also the most often cited for pets – Psittacidae (*Eupsittula cactorum*, n = 11), Corvidae (*Cyanocorax cyanopogon*, n = 5), Thraupidae (*Paroaria dominicana*, n = 8; *Sicalis flaveola*, n = 12) and Cardinalidae (*Cyanoloxia brissonii*, n = 4). A selection of the bird species used for game and captivity (some of which were observed in the residences of some of the interviewees) are shown in Figure 2.

Another relevant finding is that species such as the Blue-fronted Parrot (*Amazona aestiva*), a parrot popular as a pet, and the Roadside hawk (*Rupornis magnirostris*) were cited as game species. In fact, the informants who reported this practice confirmed that they would hunt as game any bird they encountered in the wild, “when we go out to hunt, we’ll kill any bird that crosses our path”.

Six of the 24 interviewees confirmed that they used five species of bird for medicinal purposes. One informant reported using the White-naped Jay (*C. cyanopogon*) (Figure 2G) as a cure for speech problems – “I used the bird to help my brother speak – he hadn’t spoken for three years”. Two specimens were captured and cooked without salt before being consumed by the patient, who “was speaking after just a few days”, according to the informant. Another informant reported that speech problems could be solved easily by removing the head of a live jay and drying it being hanging it from the child’s neck. Two informants referred to the use of an infusion of the feathers of the Yellow-legged Tinamou (*Crypturellus noctivagus*) as a cure for snakebite, in which case, they “burn the feathers, take the ash and put it in some drinking water”.

The Smooth-billed Ani (*Crotophaga ani*) (Figure 2I) is used for the treatment of “chronic cough”, for which the bird is also consumed after being cooked without salt. Another way of using this species for the treatment of asthma, according to a different informant, is to roast the animal until it has turned into “powder” and feed this substance to the patient without their knowledge.

Both species of tinamous (*Crypturellus parvirostris* and *Crypturellus tataupa*) were cited by one informant for the treatment of children with walking difficulties. The treatment consisted of cutting off the bird’s feet and drying them before hanging them around the neck of the child. This treatment was explained by the fact that the chicks of these species are able to run as soon as they hatch.

## Discussion

In a study of the illegal trade in wild birds in the semi-arid zone of the Brazilian state of Paraíba, Barbosa et al. [8] recorded a preference for the raised bird with the most attractive songs and coloration. This preference was also observed in the present study, where the majority of the informants interviewed at Macaúba confirmed that the plumage and song are the characteristics that most attract their attention in wild birds, supporting the hypothesis that the selection of species by the informants was motivated primarily by attributes considered by them to be attractive. The potential of the birds as game species is also taken into consideration, and the species with the highest UV scores, which have a high hunting potential, were the most frequently cited overall by the informants.

All the informants from Macaúba indicated that a bird’s song was the characteristic that most attracted their attention, followed by the color of the plumage, indicating that they appreciate these attributes. Despite this, hunting game to supplement the diet is the primary use of birds by the informants, a situation similar to that found in previous studies, such as those of Bezerra et al. [2], Mendonça et al. [4], Barbosa et al. [8] and Souza and Alves [17]. However, this is the opposite of the pattern found by Albuquerque et al. [35] and Alves et al. [36], who recorded raised birds in captivity as the primary use of the bird fauna in other areas of the Brazilian semi-arid zone, which may be related to the raised for food to complement the diet at the site of the present study. An additional characteristic of the present study was the captivity of songbirds and other species as personal pets rather than for commercial reasons or the illegal trade in wild animals.

One of the game species cited in the present study, with high use value, was *Leptotila verreauxi*. Similar bird (*Leptotila rufaxilla*) was also reported as a game species in Sierra Nanchititla, Mexico [9], indicating similarities in the preference for game species between countries.

The informants from Macaúba presented a marked preference for certain types of species for pets, in particular psittacids, corvids, thraupids. Others such as tinamids, columbids, and tyrannids were used for food. These findings are similar to those of Bezerra et al. [2], Mendonça et al. [4], Barbosa et al. [8], Pagano et al. [26] and Santos-Fita and Costa-Neto [37]. However, one family cited as use for food by many informants (Thamnophilidae) has not been recorded in previous ethno-ornithological studies, and the hunting of these species may be a local tradition.

Only three specialists reported using birds for medicinal purposes, possibly because these animals are rarely used for this purpose in general. However, this practice may represent a local tradition, handed down over the generations, as observed by Bezerra et al. [21]. The therapeutic use of one of the three species cited in the present study – *C. cyanopogon* – has also been recorded at a number of other Brazilian localities [14, 24, 38]. In Bahia state, Costa-Neto [39] recorded the medicinal use of all three species cited in the present study. In two of these cases, only the feathers were exploited – those of *C. cyanopongon* for neurological problems, and of *C. noctivagus* for cases of epilepsy. The smooth-billed anu (*C. ani*) is used in two ways – the bird is eaten whole as a treatment for morning sickness and its blood is taken as a cure for asthma (unspecified). Ferreira et al. [38] also recorded the use of *C. ani* feathers for the treatment of asthma in Crato, Ceará.

The results of the present study not only corroborate those of these previous studies, but provide evidence of variations in practices. Rather than using the feathers, for example, *C. cyanopogon* was cooked without salt and eaten whole for the treatment of a speech impediment, whereas *C. ani* was eaten whole as a cure for “chronic cough”. The feathers of *C. noctivagus* were used at Macaúba, but as a treatment for snakebite. This study represents the first published report of the zootherapeutic use of *C. parvirostris* and *C. tataupa* for the treatment of children with walking difficulties. Importantly, much of the use of birds as zooterapics is related to popular belief the region.

## Conclusions

The results of the present study indicated that songbirds and other species with colorful plumage most attracted the attention of local residents, and are frequently captured for pets. However, game species are the primary objective of hunting activities.

The species targeted most frequently both for hutting and pets were the most attractive in terms of their plumage, song and/or meat. A number of the species identified in this study were recorded for these uses for the first time.

The evidence recorded for the medicinal use of birds in the Brazilian semi-arid zone is an important contribution to the study of zootherapy, which has important implications for conservation biology, public health policies, and the sustainable management of natural resources. Obviously, further studies are required in order to determine whether the treatments do in fact have any medicinal properties. If there is no scientific proof, the evidence should be used to discourage the exploitation of the species for this purpose.
